# Nutrient intake in low-carbohydrate diets in comparison to the 2020–2025 Dietary Guidelines for Americans: a cross-sectional study

**DOI:** 10.1017/S0007114522001908

**Published:** 2023-03-28

**Authors:** Maximilian Andreas Storz, Alvaro Luis Ronco

**Affiliations:** 1 Centre for Complementary Medicine, Department of Internal Medicine II, Faculty of Medicine, Freiburg University Hospital, 79106 Freiburg, Germany; 2 Unit of Oncology and Radiotherapy, Pereira Rossell Women’s Hospital, Bvard. Artigas 1590, 11600 Montevideo, Uruguay; 3 School of Medicine, CLAEH University, Prado and Salt Lake, 20100 Maldonado, Uruguay; 4 Biomedical Sciences Center, University of Montevideo, Puntas de Santiago 1604, 11500 Montevideo, Uruguay

**Keywords:** Nutrition, Low-Carbohydrate Diet, Macronutrients, Minerals, Vitamins, Deficiencies

## Abstract

The percentage of US adults following low-carbohydrate diets (LCD) doubled in the last decade. Some researchers observed this trend with concern and highlighted the potential for nutritional deficiencies and impaired overall diet quality with LCD. The present study investigated nutrient intake in a nationally representative sample of 307 US adults following an LCD. Using cross-sectional data from the National Health and Nutrition Examination Surveys, we compared nutrient intake profiles in said individuals with the daily nutritional goals specified in the current 2020–2025 Dietary Guidelines for Americans (DGA). Results were then compared with the general population consuming a standard American diet. Almost 57 % of low-carbohydrate dieters were female, and the mean age was 48·67 (1·35) years. Individuals consuming LCD exceeded the recommendations for saturated fat, total lipid and sodium intake (both sexes). An insufficient intake was observed for fibre, Mg, potassium and several other vitamins (vitamins A, E, D in both sexes as well as vitamin C in men and folate in women). Neither men nor women met the recommendations for fibre intake. A comparable picture was found for the general population. The potentially insufficient intake of several essential nutrients in LCD warrants consideration and a careful assessment with regard to the current DGA.

Low-carbohydrate diets (LCD) are a matter of controversy^(1)^, but have recently been promoted for a variety of health conditions, including type 2 diabetes and obesity^(2)^. A reduced overall intake in carbohydrates is common to all low carbohydrate approaches, but a clear consensus on what defines an LCD is missing^(3)^. Traditionally, LCD derive < 26 % of total energy from carbohydrates or include < 130 g of carbohydrates per day (Fig. 1)^(3)^.

**Fig. 1.**
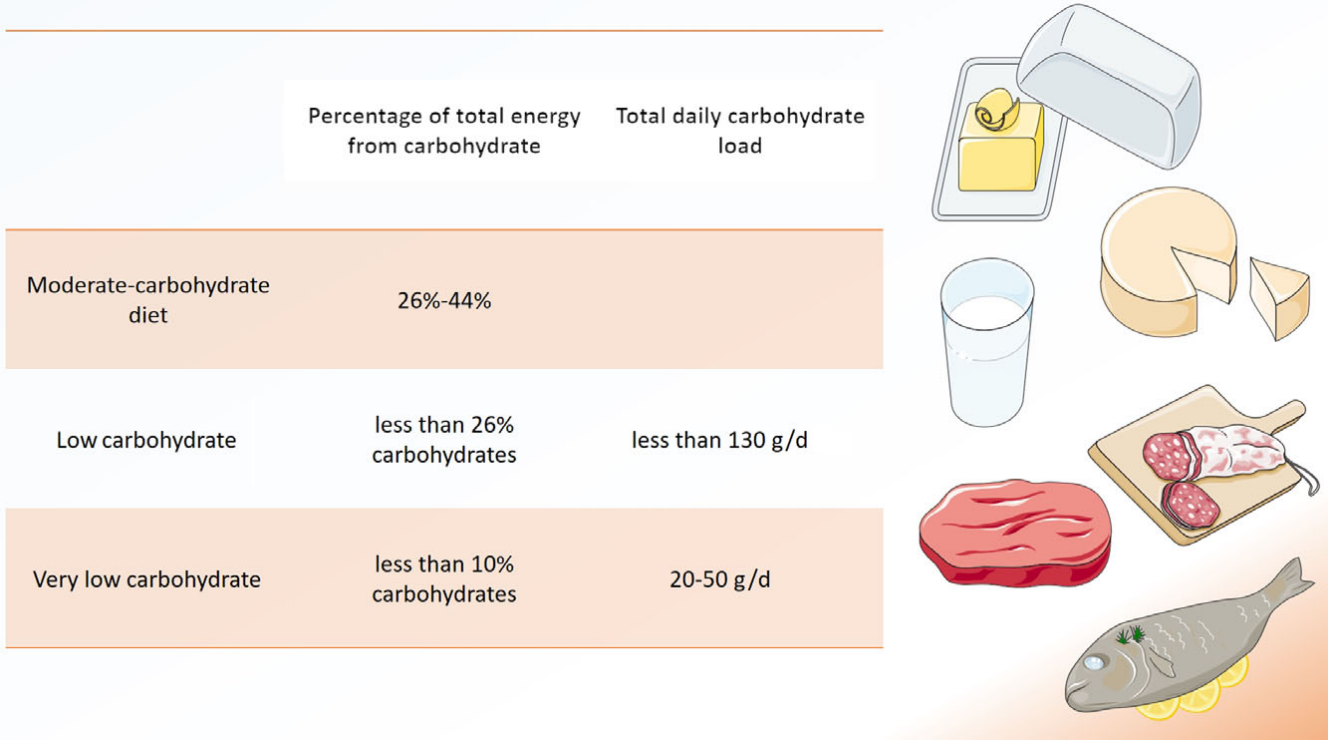
Definitions of ‘low-carbohydrate diet’. Several definitions for low-carbohydrate diets (LCD) exist, either based on percentage of total energy from carbohydrate or based on total daily carbohydrate load. Modified from Oh et al.(3). Modified from Servier Medical Art database by Servier (www.smart.servier.com Creative Commons 3.0).

Very LCD derive < 10 % of total energy from carbohydrates^(3)^. A special form of very LCD is the ketogenic diet, which generally limits carbohydrate intake to 50 g/d^(4)^. The so-called ‘classic’ ketogenic diet is characterised by a 4:1 ratio of fat to carbohydrates^(2,4)^. As such, fat may provide more than 90 % of total energy intake^(4)^.

Unfortunately, the large heterogeneity in definitions of LCD makes comparison of clinical trials using that particular dietary approach difficult. Furthermore, LCD often differ in their diet composition (e.g. with regard to carbohydrate quality), which may also lead to conflicting results in clinical studies^(5)^.

While occasionally recommended for weight loss^(6)^, reliable long-term data indicating sustainable dietary effects are scarce^(1)^. One of the frequently mentioned potential short-term benefits of LCD is improved glycaemic control in individuals with type 1 diabetes and in individuals with overweight^(7,8)^. Ketogenic diets are probably best known for their potential benefits in children with drug-resistant epilepsy, although evidence for their effectiveness in adults remains uncertain^(9)^.

Yet, there are various studies which associated carbohydrate-restricted dietary patterns with adverse health outcomes, including (but not limited to) an increased risk of type 2 diabetes in men^(10)^, weight gain^(11)^ and cardiac arrhythmias^(12)^. LCD have also been associated with an increased overall and cause-specific mortality in two large independent studies^(13,14)^.

Nevertheless, the percentage of US adults following an LCD more than doubled between 2007/2008 and 2017/2018, and increased from 0·9 % to 2·2 %^(15)^. Some researchers observed this trend with great concern and highlighted potential nutritional deficiencies and impaired overall diet quality when following LCD^(2,16)^.

LCD are typically high in saturated fat and cholesterol^(17)^, while often low in certain vitamins (A, E and B6) and micronutrients (such as Mg and potassium)^(16,17)^. Freedman, King and Kennedy emphasised that high-fat, low-carbohydrate diets are nutritionally inadequate^(16)^, whereas other authors provided opposite results^(18)^. Despite these persistent controversies, positive media support for low-carbohydrate-high-fat diets may be tempting for many individuals^(11)^, who are often unaware of potential nutritional deficiencies when restricting carbohydrates. Unawareness may also be present with regard to the excessive intake of potentially harmful nutrients (e.g. saturated fat).

To raise awareness, the present study sought to re-visit diet quality and nutrient intake in a nationally representative sample of US adults following an LCD. Using data from the National Health and Nutrition Examination Surveys (NHANES), we compared nutrient intake profiles of self-identified low-carbohydrate dieters with the daily nutritional goals (DNG) specified in the current 2020–2025 Dietary Guidelines for Americans (DGA)^(19)^. We sought to investigate for which nutrients the DNG were met, and whether an insufficient (or excessive) nutrient intake occurred. Moreover, we compared the intake profiles of LCD consumers with the US general population denying a special diet.

## Materials and methods

### The Health and Nutrition Examination Surveys

The NHANES is a cross-sectional and nationally representative US-based survey designed to assess the health and nutritional status of noninstitutionalised adults and children in the USA^(15,20)^. NHANES is conducted by the National Center for Health Statistics, which is part of the Centers for Disease Control and Prevention. The survey examines a sample of approximately 5000 persons per annum and includes demographic, socio-economic, dietary, and health-related interview questions.

Interviews are standardised and conducted in participants’ homes^(20)^. Health measurements are performed in specially equipped and designed mobile examination centres, which travel to locations throughout the entire country^(15,20)^. A detailed description of the NHANES may be obtained from the official NHANES homepage^(21)^.

### Population

We merged and appended multiple NHANES modules, including the dietary interview module and the demographics public release file^(22,23)^, which contains demographic data (age, sex, race/ethnicity, marital status, etc.) and the sampling weights. The dietary interview component included detailed dietary intake information from NHANES participants. We obtained estimates of energy and nutrient intake from the first day of the dietary recall and extracted information on all nutrients, vitamins and minerals included in the DNG Table A1–2 in the 2020–2025 DGA^(19)^. To calculate the percentage of total energy from each macronutrient, we used Atwater’s values for the metabolisable energy of macronutrients^(24,25)^.

As part of the dietary interview, participants were asked ‘Are you currently on any kind of diet, either to lose weight or for some other health-related reason?’^(22)^. Those who answered with ‘yes’ were subsequently asked ‘What kind of diet are you on?’. No list of diets or standardised definitions were provided. Responses were categorised to ‘weight loss or low energy diets’, ‘low fat/low cholesterol diet’, ‘low salt/low sodium diet’, ‘sugar-free/low-sugar diet’, ‘low-fibre diet’, ‘high-fibre diet’, ‘diabetic diet’, ‘low-carbohydrate diet’, ‘weight gain/muscle building diet’, ‘high protein diet’ or ‘other special diet’. From 2009 onwards, additional diets (e.g. ‘gluten-free or celiac diet’) were added. For the present study, we investigated nutrient intake in those individuals following an LCD. In addition, we investigated nutrient intake in the general population that denied consumption of a special diet (answering ‘no’ to the aforementioned question on special diets) and consumed the average American diet. Nutrient intake profiles of both groups were then descriptively compared with the DNG in the current DGA (without statistical calculations).

### Dietary Guidelines for Americans

The DGA is the cornerstone of US Federal nutrition policy and nutrition education activities^(26)^. A major aim of the DGA is to provide food-based recommendations to promote health and to help prevent diet-related diseases. The DGA is published jointly by the US Department of Health and Human Services and the US Department of Agriculture every 5 years.

Designed for nutrition and health professionals to support all individuals consume a healthy, nutritionally adequate diet, the current DGA encompasses 164 pages. Given the many dietary components of public health concern for the general US population (potassium, dietary fibre, vitamin D, etc.), the DGA also include age–sex-specific nutritional goals which can be found in the appendix (page 131)^(19)^.

DNG are available for both sexes and stratified by age groups (e.g. 19–30 years, 31–50 years and 51+ years). We made use of this classification and descriptively compared nutrient intake in LCD consumers (and the general population denying a special diet) with the DNG stratified by age–sex groups. The case number of individuals aged 18 years or younger following an LCD was severely limited (< 30 individuals). Thus only individuals aged 19 years or older were considered eligible for this study.

The DNG in the current DGA stem from various sources. Sources and concepts include adequate intake, Acceptable Macronutrient Distribution Range (AMDR), Chronic Disease Reduction Level, DGA and the Recommended Dietary Allowance (RDA). The adequate intake is a dietary recommendation used when there is not enough available scientific data to calculate an average nutrient requirement^(27)^. An adequate intake is the ‘average nutrient level consumed daily by a typical healthy population that is assumed to be adequate for the population’s needs’. RDA are the ‘levels of intake of essential nutrients that […] are judged by the Food and Nutrition Board to be adequate to meet the known nutrient needs of practically all healthy persons’^(28)^. The Chronic Disease Reduction Level represents the lowest level of intake of a nutrient for which there is sufficient evidence to characterise a chronic disease risk reduction. In this analysis, it is used only for sodium and reflects the intake above which intake reduction is expected to reduce chronic disease risk within an apparently healthy population^(29)^. The AMDR describes recommendations for macronutrient intake in the context of a complete diet and expresses intake recommendations as a percentage of total energetic intake^(30)^. Epidemiological evidence suggested that consumption within these ranges may also play a role in reducing risk of chronic diseases^(31)^.

Our analysis covered all nutrients, vitamins and minerals included in the DNG Table A1–2 of the current DGA (2020–2025)^(19)^. Nutrients included carbohydrate, protein and fat (reported in g/d and in %/total kcal intake), saturated fat intake, linoleic acid intake and linolenic acid. Minerals included Ca, iron, Mg, phosphorus, potassium, sodium and Zn. Vitamins included vitamins A, E, D, C, B_1_, B_2_, niacin, B_6_, B_12_, K and folate. Finally, we analysed fibre intake in all groups.

### Statistics

The NHANES sample is selected through a complex, multistage probability design^(15)^, and we used the ‘svyset’ and ‘svy’ commands for all statistical procedures to properly account for population weights and the NHANES survey design characteristics. We conducted all statistical analyses with STATA software version 14 (StataCorp.)^(32)^. We performed unconditional subclass analyses (preserving the main survey design and providing larger standard errors) to estimate nutrient intake in self-identified LCD followers^(33)^. To increase the sample size for analyses stratified by population subgroups, we appended six NHANES survey cycles (2007–2008, 2009–2010, 2011–2012, 2013–2014, 2015–2016 and 2017–2018). We only included individuals with a full data set.

Normally distributed variables were described with their mean and standard error in parentheses. For categorical variables, we reported the number of observations (*n*) as well as weighted proportions (with their corresponding standard error) in parenthesis.

To facilitate comparison between estimated nutrient intake in low-carbohydrate dieters and the recommendations found in the DGA, we employed colour coding for all tables. Green colour indicates that the DNG were met, whereas red colour indicates a violation of the recommendations. This could be either an excessive intake (e.g. excessive sodium intake) or an insufficient intake (for example, a lack of potassium in diet), as indicated by the arrow direction. For energy intake (in kcal/d), orange colour coding was used. All comparisons were performed in an entirely descriptive way, without testing for statistical significance.

## Results

### Sample characteristics

After removal of individuals aged 18 years or younger and after removal of individuals with an incomplete data set (*n* 27 in the LCD group and *n* 19 086 in the general population), the analysed sample included *n* 307 individuals on a LCD. This may be extrapolated to represent 3 089 597 US Americans on an LCD. Sample characteristics are presented in Table 1.

**Table 1.** Sample characteristics (Numbers and percentages; standard errors)

	Individuals on an LCD			General population		
	Number of observations (*n*)	Weighted proportion (%)	se	Number of observations (*n*)	Weighted proportion (%)	se
Age						
19–30 years	*n* 46	17·11	3·15	*n* 5729	23·34	0·66
31–50 years	*n* 116	33·51	3·82	*n* 8866	35·55	0·62
51 years and older	*n* 145	49·39	4·80	*n* 12 198	41·11	0·63
Sex						
Male	*n* 119	42·72	4·18	*n* 13 431	49·76	0·38
Female	*n* 188	57·28	4·18	*n* 13 362	50·24	0·38
Race/Ethnicity						
Mexican American	*n* 32	4·81	1·14	*n* 4111	8·96	0·78
Other Hispanic	*n* 21	3·10	0·98	*n* 2770	5·91	0·47
Non-Hispanic White	*n* 158	74·41	2·85	*n* 11 001	65·51	1·48
Non-Hispanic Black	*n* 43	5·39	1·02	*n* 5852	11·67	0·78
Other Race - Including Multi-Racial	*n* 53	12·29	2·14	*n* 3059	7·95	0·47

LCD, low-carbohydrate diets.

The sample included *n* 307 individuals on a low-carbohydrate diet and *n* 26 793 denying a special diet.

Mean age of the entire LCD cohort was 48·67 (1·35) years. Males who consumed an LCD were slightly older (48·71 (1·58) years) than females (48·64 (1·58) years). Our data also suggested that LCD were more popular among females. More than 74 % of the LCD cohort were of Non-Hispanic White origin. The general population denying any special diet included 26 793 individuals, which may be extrapolated to represent 194 297 552 US Americans. Sample characteristics may be obtained from Table 1.

### Macronutrient and fibre intake

Table 2 compares (macro-)nutrient and fibre intake in males following an LCD with the DNG in the 2020–2025 DGA, stratified by age group. In a similar style, Table 3 compares nutrient intake and fibre intake in females following an LCD with the current DGA.

**Table 2.**
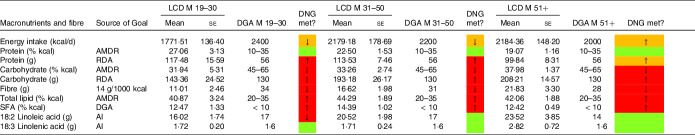
Macronutrient and fibre intake in males following a low-carbohydrate diets (LCD) compared with the daily nutritional goals (DNG) in the 2020–2025 Dietary Guidelines for Americans (DGA) stratified by age group (Mean values and standard errors of the mean)

AMDR, Acceptable Macronutrient Distribution Range; RDA, Recommended Dietary Allowance; AI, adequate intake; based on^(19)^.

**Table 3.**
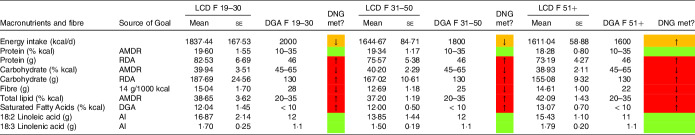
Macronutrient and fibre intake in females following a low-carbohydrate diet (LCD) compared with the daily nutritional goals (DNG) in the 2020–2025 Dietary Guidelines for Americans (DGA) stratified by age group (Mean values and standard errors of the mean)

AMDR, Acceptable Macronutrient Distribution Range; RDA, Recommended Dietary Allowance; AI, adequate intake; based on^(19)^.

Males on an LCD had a higher energy intake than females on an LCD, except in the group aged 19–30 years. Energy intake was below the recommended daily nutritional goal in both sexes, except in males and females aged 51 years or older (Table 2).

Individuals consuming an LCD exceeded the daily nutritional goals for protein and fat (in g/d). Males aged 31–50 years consumed more than twice as much total protein (113·53 (7·46) g) as recommended (56 g). Both sexes obtained < 41 % of total energy from carbohydrates. By the definition of Oh, Gilani, and Uppaluri, both groups moderately restricted carbohydrates (3). The lowest carbohydrate intake (expressed as a percentage of total energy intake) was observed in males aged 19–30 years (31·94 %). Carbohydrate restriction was generally less pronounced in females (39·94 %, 40·20 % and 38·92 %) as compared with men (31·94 %, 33·26 % and 37·98 %).

Males aged 31–50 years and 51+ years consumed significantly more total fat (106·05 (8·61) g/d and 103·34 (10·06) g/d, respectively) than females (69·48 (4·96) g/d and 76·43 (4·68) g/d, respectively). In participants aged 19–30 years, females consumed slightly more total fat (81·41 (11·97) g/d) than males (80·67 (8·58) g/d). This pattern was also found with regard to saturated fat intake, monounsaturated fat intake and polyunsaturated fat intake. Males aged 31–50 years consumed, on average, 34·39 (3·28) g of saturated fat per day, whereas males aged 51+ years consumed 30·40 (2·52) g/d. In females, average consumption of saturated fat was lower in both age groups (22·38 (1·86) and 24·12 (1·94) g/d, respectively). Saturated fat intake (expressed as a percentage of total kcals) in both sexes exceeded the recommendations in the current DGA. Moreover, our data suggest that both sexes failed to meet the daily nutritional goals for fibre. Total fibre intake in g/d is reported in Tables 2–5. The lowest fibre intake was found in males aged 19–30 years on an LCD (11·01 g (2·46)). Notably, intake of 18:2 linoleic acid was also below the recommendations in males aged 19–30 years adhering to an LCD.

**Table 4.**
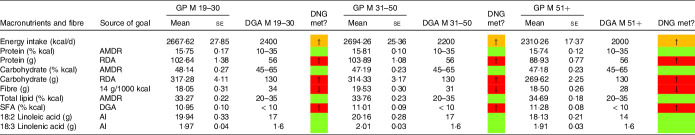
Macronutrient and fibre intake in males denying a special diet compared with the daily nutritional goals (DNG) in the 2020–2025 Dietary Guidelines for Americans (DGA) stratified by age group (Mean values and standard errors of the mean)

AMDR, Acceptable Macronutrient Distribution Range; RDA, Recommended Dietary Allowance; AI , adequate intake; based on^(19)^.

**Table 5.**
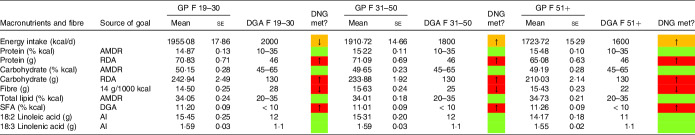
Macronutrient and fibre intake in females denying a special diet compared with the daily nutritional goals (DNG) in the 2020–2025 Dietary Guidelines for Americans (DGA) stratified by age group (Mean values and standard errors of the mean)

AMDR, Acceptable Macronutrient Distribution Range; RDA, Recommended Dietary Allowance; AI, adequate intake; based on^(19)^.

In a similar style, Tables 4 and 5 display macronutrient and fibre intake in males and females in the general population denying a special diet. When comparing energy intake in males on an LCD with males in the general population, Tables 2 and 4 suggest substantial differences. In all three age groups, energy intake in LCD consumers was well below the intake in the general population. Carbohydrate intake (expressed as a percentage of total energy intake) in the general population was consistently within the acceptable macronutrient distribution range. The same applied for total lipid intake (AMDR, Table 4). The male general population exceeded the DGA recommendation for SFA intake; however, compared with LCD consumers, the difference was less pronounced. When comparing females denying a special diet (Table 5) with females on an LCD diet (Table 3), we observed a comparable picture. Across all three age groups, females in the general population met the recommendations for carbohydrate intake (AMDR) and total lipid intake (AMDR).

### Mineral and vitamin intake

In a similar style, Tables 6–9 display mineral and vitamin intake, across all age categories. Males on an LCD did not meet the recommendations for potassium intake (Table 6) and Mg intake (except the group aged 19–30 years). Sodium intake exceeded the DGA recommendations, particularly in males aged 31–50 years. Ca intake in males aged 51+ years was also below the RDA in the current DGA. A comparable picture was found in the general population denying a special diet (Table 8).

**Table 6.**
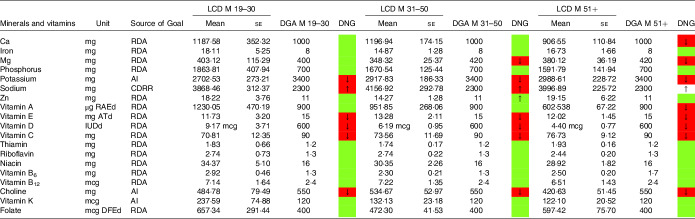
Mineral and vitamin intake in males following low-carbohydrate diets (LCD) compared with the daily nutritional goals (DNG) in the 2020–2025 Dietary Guidelines for Americans (DGA) stratified by age group (Mean values and standard errors of the mean)

CDRR, Chronic Disease Reduction Level; RDA, Recommended Dietary Allowance; AI, adequate intake; based on^(19)^.

**Table 7.**
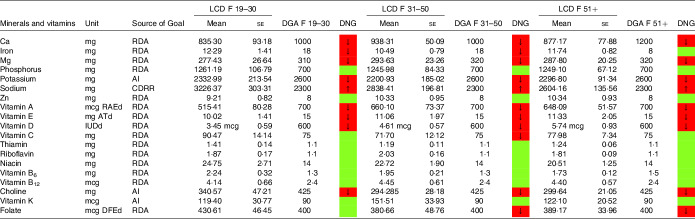
Mineral and vitamin intake in females following low-carbohydrate diets (LCD) compared with the daily nutritional goals (DNG) in the 2020–2025 Dietary Guidelines for Americans (DGA) stratified by age group (Mean values and standard errors of the mean)

CDRR, Chronic Disease Reduction Level; RDA, Recommended Dietary Allowance; AI, adequate intake; based on^(19)^.

**Table 8.**
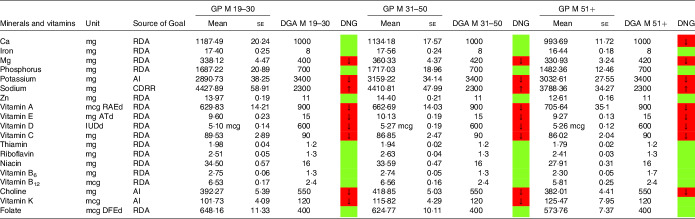
Mineral and vitamin intake in males denying a special diet compared with the daily nutritional goals (DNG) in the 2020–2025 Dietary Guidelines for Americans (DGA) stratified by age group (Mean values and standard errors of the mean)

CDRR, Chronic Disease Reduction Level; RDA, Recommended Dietary Allowance; AI, adequate intake; based on^(19)^.

**Table 9.**
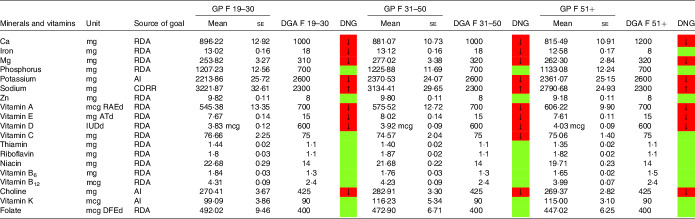
Mineral and vitamin intake in females denying a special diet compared with the daily nutritional goals (DNG) in the 2020–2025 Dietary Guidelines for Americans (DGA) stratified by age group (Mean values and standard errors of the mean)

CDRR, Chronic Disease Reduction Level; RDA, Recommended Dietary Allowance; AI, adequate intake; based on^(19)^.

Females aged 19–50 years on an LCD did not meet the recommendations for iron (Table 7). Intakes below the recommendations were also found for Ca (particularly in female LCD consumers aged 51+ years) and Mg. Again, a comparable picture was found in the general population (Table 9).

Moreover, both sexes failed to meet the recommendations for vitamins A and E. Males on an LCD also failed the recommendations for vitamin C (Table 6) across all age groups. A comparable picture was found in females aged 31–50 years on an LCD.

Notably, vitamin A intake was below the RDA in males aged 51+ years on an LCD (Table 6). In females on an LCD, this pattern was observed across in age categories. For intakes in the general population, the reader is referred to Tables 8 and 9.

NHANES reports vitamin D intake in μg/d (see Tables 6–9). One μg of vitamin D equals 40 IE, and as such both sexes were well below the RDA of 600 IE. An insufficient intake was also observed with regard to choline (in both sexes) and folate (in females reporting an LCD aged 31+ years only).

Intake of minerals and vitamins in the general population denying a special diet stratified by age groups may be obtained from Tables 8 and 9. Notable differences in males were found with regard to vitamin K and vitamin A. In females, there were notable differences with regard to folate,

## Discussion

The present study sought to investigate diet quality and nutrient intake in a nationally representative sample of US adults following an LCD. Individuals on LCD exceeded the recommendations for SFA intake, total lipid intake and sodium intake (both sexes). An insufficient intake was observed for fibre, Mg, potassium and several other vitamins (vitamins A, E, D in both sexes as well as vitamin C in men and folate in women aged 31 years or older). Our findings are of great concern, given the growing interest in LCD in the US general population within the last decade^(15)^.

The ingestion of excessive amounts of SFA (as observed in individuals on an LCD in our study but also in the general population denying a special diet) is considered to be a risk factor for CVD, insulin resistance, dyslipidaemia and obesity^(34)^. A high intake of SFA favours a pro-inflammatory status that contributes to the development of insulin resistance and exerts lipotoxic effects on human tissues^(34,35)^. Furthermore, a high SFA intake has been associated with diabetes^(36)^, coronary heart disease^(37)^, hepatic and visceral fat storage^(38)^ and cognitive decline^(39)^. A high intake of saturated or trans-unsaturated (hydrogenated) fats has also been associated with an increased risk for Alzheimer disease^(40)^.

As such, researchers emphasised that dietary recommendations should continue to focus on replacing total saturated fat with more healthy sources of energy^(37)^. This has recently been confirmed by the WHO, which recommends limiting SFA intake to < 10 % of total energy intake^(41)^. The type and proportion of dietary fatty acids should also be considered. An increased intake of PUFA might attenuate the pro-inflammatory effects of a high SFA intake, in particular when taking *n*-3 PUFA into consideration^(42)^. On the other hand, reduced levels of vitamins A, C and E may enhance oxidative stress, and within such a context PUFA could be even more prone to oxidation (and thus favour inflammation)^(43)^. As such, it is advisable to reduce SFA intake overall.

Apart from not meeting the recommendations for SFA, individuals on an LCD in our sample also demonstrated an excessive sodium intake. Excess dietary sodium has been linked to elevations in blood pressure and other health repercussion^(44)^. A reduced sodium intake, in contrast, reduces blood pressure in adults and has also been associated with a reduced risk of stroke and fatal coronary heart disease^(45)^. In light of these findings, sodium intake in LCD consumers in our cohort appears of particular concern. The reservation must be made, however, that sodium intake in the general population consuming a Western diet showed a comparable picture.

Another point worth mentioning is the low fibre intake in our sample (both groups). Fibre-deficient diets have been associated with colon cancer and other bowel diseases as well as with obesity and type 2 diabetes^(32,46)^. Increased consumption of fibre-rich plant-based foods (up to a dietary fibre intake of 50 g/d) has been suggested as a potential strategy to extend lifespan and to improve the quality of the years gained by reducing the effects of diseases associated with high-income lifestyles^(46)^. Of note, LCD eaters in our sample did not meet the Institute of Medicine’s recommendations for fibre intake (suggesting an AI of 14 g/1000 kcal per day), highlighting once more the great risk for fibre deficiency with an LCD^(47)^. Again, the reservation must be made that fibre deficiency is a nutritional public health concern in the USA, as reflected by the intake data in the general population on a Western diet in our sample.

Additional deficiencies worth discussing include Mg, potassium and vitamins A, E and C (in males only). Vitamin A deficiency can lead to a series of ocular and dermatological symptoms and anaemia^(48,49)^. It has also been associated with a weak resistance to infection, which can increase the severity of infectious diseases and the risk of death^(48)^. The recommended dietary allowance (RDA) of vitamin A in the DGA in healthy adults is 900 μg/d for men and 700 μg/d for women. Individuals on an LCD (females in all age groups and males aged 51 years or older) in our sample failed to reach these targets. The same applies for vitamin E, where deficiencies have been associated with ataxia, neuropathy, anaemia and other health conditions^(50)^.

Many issues regarding benefits and risks of LCD remain controversial or unresolved^(51)^. Some authors emphasised that advocates of carbohydrate restriction are often in open disagreement with nutritional authorities^(52)^. A prominent example is Sweden, where advocates of LCD dedicated themselves to achieving an overwhelming public presence in the propagation of simplified accounts of dietary science^(53)^. During these debates, potential health risks of LCD have often been pushed into the background.

The present study sought to re-visit diet quality and nutrient intake in a nationally representative sample of US adults following an LCD. By investigating reliable estimates of nutrient intake in LCD followers, the authors aim was to compare *actual* nutrient intake in low-carbohydrate eaters with up-to-date established dietary guidelines (DGA). This simple (but nationally representative) comparison revealed a series of nutrients of concern and emphasised once more that long-term adherence to LCD could potentially result in nutritional deficiencies^(2)^. Our findings are in accordance with previous studies and reviews, summarising potential deficiencies occurring upon consumption of an LCD. As suggested by Freedmann, King and Kennedy, we also observed a low intake in vitamins E, A, folate, Ca, Mg, potassium and dietary fibre^(16)^. Our findings also confirm the aforementioned study with regard to the high total fat and saturated fat intake.

Monitoring (and supplementation) of those critical nutrients in LCD consumers might thus be warranted. Nevertheless, our results should not distract from the fact that we observed a comparable picture in the general population. The current DGA emphasise many nutrients of public health concern (fibre, Ca, etc.) and our data somewhat confirm that^(19)^.

Although cross-sectional (and thus with inherent limitations), our data allow for important descriptive insights into nutrient intake in LCD consumers. The overall picture is somewhat comparable to the general population consuming a typical Western diet and denying a special diet. An insufficient intake for many critical nutrients was observed in both groups, and the key features (e.g. the insufficient folate intake in females reporting an LCD) have been described in the Results section.

The present study draws upon a number of additional strengths. We present a nationally representative and large data set from the National Health and Nutrition Examination Survey with 307 individuals on an LCD. To the best of our knowledge, we also present the first comparison of nutrient intake in this group with the current DGA. Our sample may be extrapolated to represent 3 148 420 US Americans on an LCD and includes all nutrients presented in the DNG Table A1–2 in the 2020–2025 DGA. An additional asset is the analysis stratified by age–sex groups in full accordance with the current DGA.

Weaknesses included the cross-sectional nature of our data and the lack of other demographic variables describing our sample. Adding additional demographic variables of interest (such as marital status or income), however, would have reduced our total sample size, and as such we refrained from that step. The same applied for anthropometric data describing our sample and for information on carbohydrate quality. Furthermore, some participants reported consumption of more than one special diet at the same time. Thus, we must acknowledge that there was a certain overlap. LCD consumers also reported concurrent adherence to the following dietary patterns: weight loss diet, *n* 43; low sugar diet, *n* 31; low sodium diet, *n* 20; diabetic diet, *n* 17; high protein diet, *n* 28 and renal diet, *n* 1 and gluten-free diet, *n* 3. Simply excluding the aforementioned participants from the analysis would have led to a significant decrease in sample size, and as such we decided against that step. Given that LCD may be used for weight loss and are naturally higher in protein intake (subsequent to a shift in macronutrient distribution), one may also argue that adherence to certain combinations of special diets (e.g. LCD and weight loss diet or LCD and high-protein diet) may not be regarded as necessarily problematic. Of note, general aspects of special diets among adults in the USA have been analysed in detail by Stierman *et al.*, and the reader is referred to their data report for additional information on that topic^(15)^. Whether LCD were prescribed by healthcare professionals (e.g. for the treatment of an underlying medical condition) or were self-initiated was not ascertainable from our data.

All comparisons were performed in a descriptive way without testing for statistical significance. The lack of weighted proportions of individuals meeting the recommended intakes in the DGA is another potential limitation of our analysis. Then again, the descriptive approach (comparing mean intakes in a large sample) also has its advantages and the employed colour coding may facilitate this process. Given that the violations for many nutrients appeared to be significant from a clinical perspective (e.g. the low fibre intake of only 12·69 g/d in female LCD consumer aged 31–50 years), we refrained from calculating additional p-values.

As with all dietary recall studies, NHANES dietary interviews are also subject to recall bias. Notably, some of the DNG found in the current DGA are meant to be met over time. Usage of a single 24-h-dietary recall to assess nutrient intake at a single point could thus be interpreted as problematic, although the recall method itself is considered reliable^(54)^. Finally, LCD status was self-reported, which may also have contributed to the aforementioned bias. The same may apply to individuals that denied consumption of a special diet; here again reporting and recall bias may not be fully excluded.

### Conclusion

Individuals following an LCD in this sample exceeded the recommendations for SFA, lipid and sodium intake while not meeting the intake recommendations for fibre, potassium and several other vitamins. Comparable intake violations were found in the general population. Given the continuously increasing interest in LCD in the US general population, our findings are of high importance. Although cross-sectional in nature, our data raise the possibility of inadequate nutrient intakes in LCD. Furthermore, given the continuously increasing interest in LCD in the US general population, we believe that our findings are important as evidence that does not support this dietary style as an entirely healthy one because of its potential deficiencies. Regular monitoring of critical nutrients (e.g. fibre, vitamins A and E, folate and iron) might thus be advisable.
